# Alternative strategies for selecting subsets of predicting SNPs by LASSO-LARS procedure

**DOI:** 10.1186/1753-6561-6-S2-S9

**Published:** 2012-05-21

**Authors:** M Graziano Usai, Antonello Carta, Sara Casu

**Affiliations:** 1 Settore Genetica e Biotecnologie, AGRIS-Sardegna, Olmedo 07040, Italy

## Abstract

**Background:**

The least absolute shrinkage and selection operator (LASSO) can be used to predict SNP effects. This operator has the desirable feature of including in the model only a subset of explanatory SNPs, which can be useful both in QTL detection and GWS studies. LASSO solutions can be obtained by the least angle regression (LARS) algorithm. The big issue with this procedure is to define the best constraint (*t*), *i.e*. the upper bound of the sum of absolute value of the SNP effects which roughly corresponds to the number of SNPs to be selected. Usai et al. (2009) dealt with this problem by a cross-validation approach and defined *t *as the average number of selected SNPs overall replications. Nevertheless, in small size populations, such estimator could give underestimated values of *t*. Here we propose two alternative ways to define *t *and compared them with the "classical" one.

**Methods:**

The first (strategy 1), was based on 1,000 cross-validations carried out by randomly splitting the reference population (2,000 individuals with performance) into two halves. The value of *t *was the number of SNPs which occurred in more than 5% of replications. The second (strategy 2), which did not use cross-validations, was based on the minimization of the Cp-type selection criterion which depends on the number of selected SNPs and the expected residual variance.

**Results:**

The size of the subset of selected SNPs was 46, 189 and 64 for the classical approach, strategy 1 and 2 respectively. Classical and strategy 2 gave similar results and indicated quite clearly the regions were QTL with additive effects were located. Strategy 1 confirmed such regions and added further positions which gave a less clear scenario. Correlation between GEBVs estimated with the three strategies and TBVs in progenies without phenotypes were 0.9237, 0.9000 and 0.9240 for classical, strategy 1 and 2 respectively.

**Conclusions:**

This suggests that the Cp-type selection criterion is a valid alternative to the cross-validations to define the best constraint for selecting subsets of predicting SNPs by LASSO-LARS procedure.

## Background

A method to estimate the SNP (Single Nucleotide Polymophism) effects would be to use the least absolute shrinkage and selection operator (LASSO) approach [1]. This operator has the desirable feature of including in the model only a subset of explanatory variables, setting to zero those that have nil effects. This agrees with the assumption that many chromosome segments will not contain QTL (Quantitative Trait Locus) and therefore have zero effect, and only few are real QTL [2]. The LASSO is a constrained version of ordinary least squares which minimizes the residual sum of squares constraining the sum of absolute values of the regression coefficients. Then the LASSO solution is the set of SNP effects that satisfy:

$$\text{min}\left\{{\overset{n}{\underset{i=1}{\sum }}\left(y_{i}-\overset{m}{\underset{j=1}{\sum }}x_{ij}\beta_{j}\right)}^{2}\right\}\;\text{subject to}\;\overset{m}{\underset{j=1}{\sum }}\left|\beta_{j}\right|\leq t\;\text{for}\;t\geq 0$$

Where *y_i _*is the phenotype of the *i^th ^*individual; *x_ij _*is the genotype of the *i^th ^*individual at the *j^th ^*marker; *β_j _*is the allelic substitution effect for the *j^th ^*marker and *t *is the constraint that allows some estimated SNP effects to be exactly zero. The challenge with implementing the LASSO approach is how to best choose the constraint parameter *(t) *which in turn depends on the size of the subset of explanatory variables, in this case the number of SNPs [3].

The LASSO problem can be solved by quadratic programming [1] or by Bayesian approaches [4]. The latter have been implemented in several genome wise selection (GWS) and QTL detection studies [5,6]. An alternative way to produce LASSO solution is a modified version of the Least Angle Regression (LARS) algorithm [7]. This procedure is a version of traditional forward selection methods which estimates the effects by successive iterations. For each iteration the SNP with the highest absolute correlation between genotypes and current residuals is added to the model. To obtain LASSO solutions the LARS procedure is modified so that either addition or subtraction of one marker to the model per iteration may occur. Usai *et al*. [8] suggested the LASSO-LARS method to estimate the marker effects for genomic selection including a cross-validation step to define the best constraint *t*. With this approach good results were obtained both in simulated and real dataset. Nonetheless one limitation of LASSO-LARS is that, as a constrained version of the ordinary least squares, it cannot estimate the effects for a number of markers larger than the number of individuals in the reference population. Thus in real data, where the reference population size is often relatively limited and for those traits affected by a large number of QTL, LASSO-LARS may not be able to predict the effect of all the QTL contributing to the total genetic variability. Moreover, by using cross-validations at each replicate the training sample where SNP effects are estimated is only a portion of the total reference population. So the number of SNPs selected at each replication will be smaller than expected and their average overall replications will underestimate the true value of the best *t*.

In this study we propose two alternative strategies to define the *t *and compared them with the classical strategy [8].

## Methods

### Data

A simulated data set of 3,220 individuals generated for the 15th QTL-MAS workshop was used. The first generation consisted in 220 founders (20 males and 200). The second generation consisted in 3,000 individuals organized in 20 sire families of 150 individuals each and 200 dam families of 15 individuals each. The dam families were nested in the sire families. The genome consisted in five chromosomes. Each chromosome was 1 Morgan long and carried 1998 SNPs evenly distributed. Genotypes were available for all the individuals. Phenotypes were available only for 2,000 progenies (1/3 of each sire and dam family) which represented the reference population. The further 1,000 progenies had genotypes but no phenotypes and represented the candidate population.

### LASSO-LARS classical strategy

At each cross-validation replication the reference population was randomly split into training (T) and validation (V) samples of equal size. This strategy corresponded to that suggested by Usai *et al*. [8] where the 50 % random splitting was chosen since it gave the lowest *t *variability and the highest accuracy of GEBV estimates. At each replication LASSO-LARS was run on T sample. At each step of the procedure the genomic breeding values (GEBV) of V was updated by the current set of SNP effects estimated on T. When the maximum of the correlation between the GEBVs and phenotypes of V was reached the LASSO-LARS was stopped and the number and the identity of the active SNPs were retained. The procedure was replicate 1,000 times. Afterwards the average number of active SNPs was taken as the best value of *t*. Moreover the SNP frequency of occurrence (*fo*) was defined as the proportion of times that a SNP was selected (i.e. with non zero effect) over all replications.

### Strategy 1

The only difference with respect to the classical strategy was that here the best *t *was defined as the number of SNPs which occurred in the cross-validations more than 5% of the times. This strategy was based on the assumption that if a SNP is selected more than expected by chance it is probable that it affects or is linked to QTL affecting the traits. The probability of a SNP to be selected by chance was estimated by permuting 10,000 times phenotypes on genotypes and by running LASSO-LARS until the best *t *estimated for classical strategy was attained. The frequency of occurrence of 5% corresponded to a probability lower than 0.0001.

### Strategy 2

In this strategy the best *t *was the number of active SNPs which minimize the value of the Cp-type selection criterion [7]. Such parameter is commonly used as a stopping rule for various forms of stepwise regression. At each *k^th ^*LASSO-LARS step Cp-type was calculated as:

$$Cp_{k}=\frac{\overset{n}{\underset{i=1}{\sum }}{\left(y_{i}-{\overset{⌢}{y}}_{i}\right)}^{2}}{\sigma_{e}^{2}}-n+2df_{k}$$

where *y_i _*and *ŷ_i _*are, respectively, the phenotype and the current predicted value of the *i^th ^*individual; *n *is the number of individual; *df *is the degree of freedom which here corresponded to the number of active SNPs and *σ_e_^2 ^*is the residual variance of the complete model, i.e. the model including all SNPs. The latter was estimated by a REML procedure running a GBLUP animal model with ASREML software [9]. The genomic relationship matrix was built as described by Hayes et al. in 2009 [10]. Since this strategy did not require cross-validations, LASSO-LARS was run on the whole reference population until the minimum Cp-type value was reached and the corresponding number of active SNPs was taken as best *t*.

### GEBV estimation

Once the best *t *were defined, LASSO-LARS was run on the whole population. For each strategy the procedure was stopped when the corresponding *t *SNPs were in the model. The estimated allelic substitution effects of the selected SNPs were used to calculate the GEBVs on the candidate population (1,000 progenies without phenotypes). The GEBV accuracy, defined as the correlation coefficients between true breeding values (TBVs) and GEBVs, and the regression coefficient of the TBVs on GEBVs were calculated for each strategy.

## Results

### Best t definition

**Figure 1:**
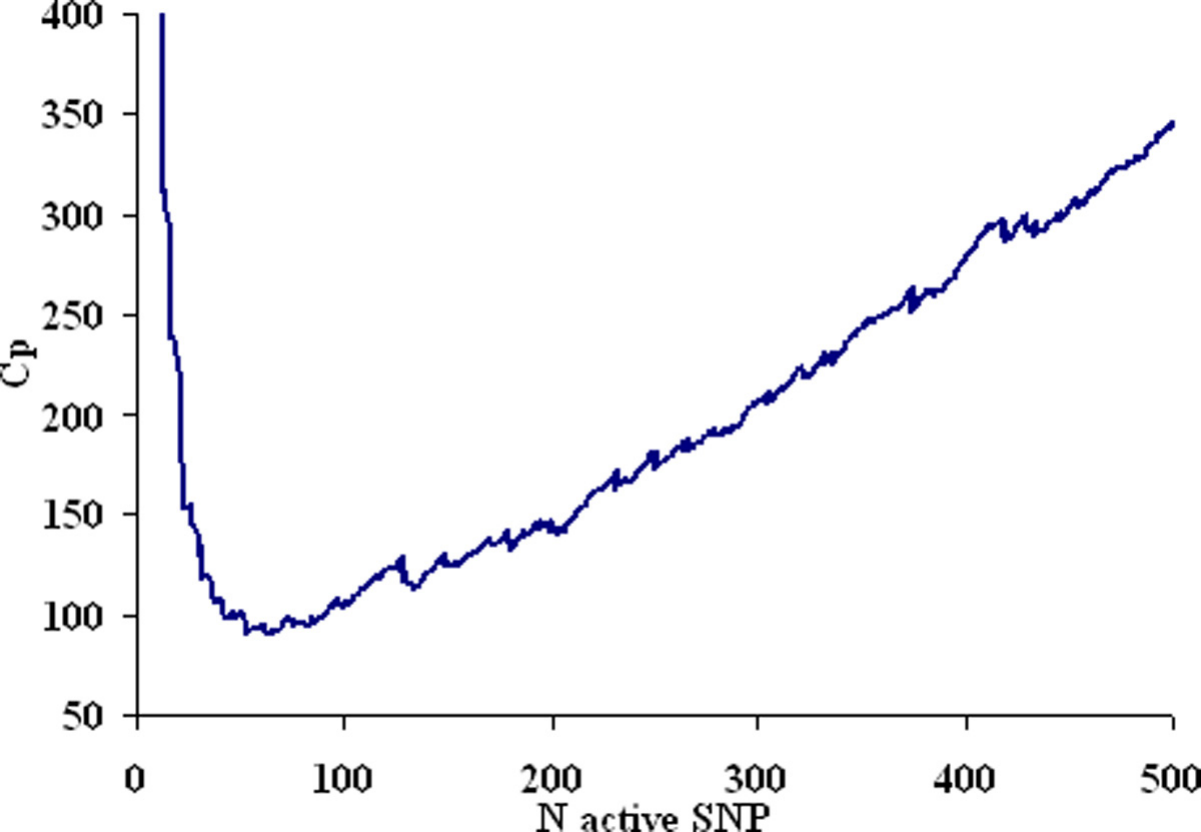
**Cp-type selection criterion profile for increasing number of active SNPs**.

The number of SNPs selected at each cross-validation replication ranged from 15 to 92 and was on average 46. This value was taken as best *t *for the classical strategy. The maximum of correlation between GEBVs and phenotypes in the validation sample was 0.493 on average and the corresponding R^2 ^was 0.298. Among the 9,990 available SNPs only 2,169 occurred at least once overall cross-validations and 189 occurred more than 5% of the times. The latter value was taken as best *t *for strategy1. Figure 1 depicts the profile of the Cp-type criterion for an increasing number of active SNPs. The minimum was reached when 64 SNPs were selected and this value was taken as best *t *for strategy2.

### QTL mapping

**Figure 2:**
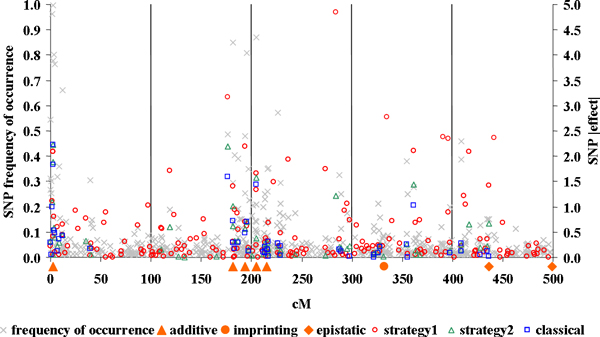
**Comparison of SNP effects estimated by classical, strategy1 and strategy2**. SNP frequency of occurrence. True QTL positions.

Figure 2 shows the absolute values of the effect of the SNPs selected by LASSO-LARS given the *t *values defined by the different strategies. The SNP *fo *in the cross-validations is also reported. The average *fo *of the SNPs selected by classical strategy was 39.8 % and ranged from 10.4 % to 99.7%. For strategy1 the *fo *was 11.7 % on average, ranging from 0 to 99.7%. In this case 107 SNPs had an *fo *lower than 5% and 16 never occurred in the cross-validations. The SNPs selected with strategy2 showed an average *fo *of 30.8% ranging from 4.4% to 99.7%. Only 3 SNPs had *fo *slightly lower then the 5% threshold. Concerning the similarity between the three SNP subsets, 39 common SNPs were selected by classical and strategy1; 44 SNPs were selected by classical and strategy2 and 55 were selected by strategy1 and strategy2. On the whole 38 SNPs were selected by all three strategies. All the three strategies identified quite clearly the five QTL with additive effects. A less clear localization was observed for the QTL with imprinted effect and the first QTL with epistatic action. None of the strategies was able to selected SNPs linked to the second epistatic QTL. These latter results were expected since LASSO-LARS only accounts for additive QTL effects. Most of the SNPs selected by classical and strategy2 were concentrated on the true QTL position. Finally the number of false detections enlarged as the SNP subset size increased and was particularly high for strategy1 where a very unclear scenario was observed.

### GEBV estimation

The candidate population GEBV accuracies corresponding to the three t estimation strategies are shown in Table 1. Not relevant differences were observed among strategies. In particular, the accuracies obtained by classical and strategy2 were very similar and both outperformed strategy1 of more than 2%. Table 1 also shows the regression of TBVs on GEBVs, indicating that both classical and strategy2 GEBVs underestimate the TBVs, whilst strategy1 gave a regression coefficient considerably closer (around 25%) to the target value 1. However, it is important to point out that some of the QTL had simulated epistatic and imprinted actions, and LASSO-LARS did not account for this.

**Table 1:** Genomic breeding value (GEBV) accuracy (r) and regression coefficient (b) of true breeding value (TBV) on GEBV for the three tested strategies

Strategy	r(TBV,GEBV)	b(TBV,GEBV)
Classical	0.9237	1.2512

Strategy1	0.9000	1.0220

Strategy2	0.9240	1.1877

## Discussion

Our results demonstrated that LASSO-LARS performs well estimating SNPs associated to QTL with additive effects. The detection of QTL with different action was rather poor. However it suggests the presence of the imprinted QTL and of the first epistatic QTL. The second epistatic QTL was neglected since LASSO-LARS just selects the SNPs which underline the main portion of the variability explained by both QTL. Concerning the choice of the best constraint for LASSO-LARS, classical and strategy2 although based on different procedures gave very similar results. This suggests that a valid estimation of the best constraint can be obtained without cross-validation with a large computing time saving. Indeed, while the cross-validation procedure took 3 hours and 35 minutes, strategy2 just took 8 seconds. Nevertheless, the current data set did not allowed to verify if the constraint estimation based on Cp-type minimization can overcome the underestimation of *t *expected with cross-validation. Thus a study based on a dataset with high ratio between number of QTL and reference population size is needed. Strategy1 seems in general the worst, since most of the selected SNPs did not correspond to those with the highest *fo*. This happened because the complementary among SNPs selected in the cross-validation was not accounted for. Indeed if two SNPs are strongly correlated and equally correlated with the phenotype, they could be alternatively selected in the cross-validation due to the random sampling. Nevertheless they explain the same portion of variance and when LASSO-LARS runs on the whole reference population only one of them is selected. In fact most of the SNPs further selected by strategy1 respect to the other two were false positives (Figure 2). The presence of many false positives leads to a lower GEBV accuracy. The regression of TBVs on GEBVs close to 1 obtained by strategy1 could be due to the higher weight given to the QTL with imprinted and epistatic effects.

## Conclusions

We conclude that the strategy based on the Cp-type selection criterion is a valid alternative to the cross-validations to define the best constraint for selecting subsets of predicting SNPs by LASSO-LARS procedure.

## List of Abbreviations used

GBLUP: Genomic Best Linear Unbiased Prediction; GEBV: Genomic Breeding Values; GWS: Genome Wise Selection; LARS: Least Angle Regression; LASSO: Least Absolute Shrinkage and Selection Operator; QTL: Quantitative Trait Locus; REML: REstricted Maximum Likelihood; SNP: Single Nucleotide Polymophism; TBV: True Breeding Value.

## Competing interests

The authors declare that they have no competing interests.

## Authors' contributions

MGU, AC and SC carried out the analyses and drafted the manuscript. All the authors have read and contributed to the final text of the manuscript.
